# Genomic and pleiotropic analyses of resting QT interval identifies novel loci and overlap with atrial electrical disorders

**DOI:** 10.1093/hmg/ddab197

**Published:** 2021-07-19

**Authors:** Stefan van Duijvenboden, Julia Ramírez, William J Young, Michele Orini, Borbala Mifsud, Andrew Tinker, Pier D Lambiase, Patricia B Munroe

**Affiliations:** Institute of Cardiovascular Science, University College London, London WC1E 6BT, UK; Clinical Pharmacology, William Harvey Research Institute, Barts and The London School of Medicine and Dentistry, Queen Mary University of London, London EC1M 6BQ, UK; Institute of Cardiovascular Science, University College London, London WC1E 6BT, UK; Clinical Pharmacology, William Harvey Research Institute, Barts and The London School of Medicine and Dentistry, Queen Mary University of London, London EC1M 6BQ, UK; Clinical Pharmacology, William Harvey Research Institute, Barts and The London School of Medicine and Dentistry, Queen Mary University of London, London EC1M 6BQ, UK; Barts Heart Centre, St Bartholomew’s Hospital, London EC1A 7BE, UK; Institute of Cardiovascular Science, University College London, London WC1E 6BT, UK; Clinical Pharmacology, William Harvey Research Institute, Barts and The London School of Medicine and Dentistry, Queen Mary University of London, London EC1M 6BQ, UK; College of Health and Life Sciences, Hamad Bin Khalifa University, Doha PO 34110, Qatar; Clinical Pharmacology, William Harvey Research Institute, Barts and The London School of Medicine and Dentistry, Queen Mary University of London, London EC1M 6BQ, UK; NIHR Barts Cardiovascular Biomedical Research Unit, Barts and The London School of Medicine and Dentistry, Queen Mary University of London, London EC1M 6BQ, UK; Institute of Cardiovascular Science, University College London, London WC1E 6BT, UK; Barts Heart Centre, St Bartholomew’s Hospital, London EC1A 7BE, UK; Clinical Pharmacology, William Harvey Research Institute, Barts and The London School of Medicine and Dentistry, Queen Mary University of London, London EC1M 6BQ, UK; NIHR Barts Cardiovascular Biomedical Research Unit, Barts and The London School of Medicine and Dentistry, Queen Mary University of London, London EC1M 6BQ, UK

## Abstract

The resting QT interval, an electrocardiographic (ECG) measure of ventricular myocardial repolarization, is a heritable risk marker of cardiovascular mortality, but the mechanisms remain incompletely understood. Previously reported candidate genes have provided insights into the regulatory mechanisms of the QT interval. However, there are still important knowledge gaps. We aimed to gain new insights by (i) providing new candidate genes, (ii) identifying pleiotropic associations with other cardiovascular traits, and (iii) scanning for sexually dimorphic genetic effects. We conducted a genome-wide association analysis for resting QT interval with ~9.8 million variants in 52 107 individuals of European ancestry without known cardiovascular disease from the UK Biobank. We identified 40 loci, 13 of which were novel, including 2 potential sex-specific loci, explaining ~11% of the trait variance. Candidate genes at novel loci were involved in myocardial structure and arrhythmogenic cardiomyopathy. Investigation of pleiotropic effects of QT interval variants using phenome-wide association analyses in 302 000 unrelated individuals from the UK Biobank and pairwise genome-wide comparisons with other ECG and cardiac imaging traits revealed genetic overlap with atrial electrical pathology. These findings provide novel insights into how abnormal myocardial repolarization and increased cardiovascular mortality may be linked.

## Introduction

The electrocardiogram (ECG) is a non-invasive, widely available and inexpensive tool to identify individuals at increased risk of cardiovascular (CV) disease. The QT interval, a marker of ventricular depolarization and repolarization, is a key measurement taken from the ECG and has considerable medical relevance as both prolongation and shortening are associated with increased CV risk in individuals with and without known CV disease (1–5). For example, each 10 ms increase in resting QT interval is associated with a 15% increased risk of CV events in the general population (5). It is also well recognized that marked prolongation and shortening of the QT interval can be caused by mutations of ion channel genes affecting depolarization or repolarization processes as observed in rare monogenic long and short QT syndromes (LQTS and SQTS) (6). While these mutations can directly affect the electrical stability of the heart and promote malignant cardiac arrhythmia (7), they only account for a very small proportion of subjects with an abnormal QT interval. The mechanisms by which an altered QT interval modulates CV risk in the absence of mutations remain incompletely understood.

The resting QT interval is a heritable trait with an estimated heritability of ~50% in twin-studies (8) and ~ 30% using common single nucleotide variants (SNVs) captured on contemporary genome-wide genotyping arrays (9). Consequently, studies have focused on identifying genetic pathways modulating the QT interval to elucidate the mechanisms by which an altered QT interval may contribute to CV risk. At present, 48 loci have been discovered for resting QT interval from genetic association studies in the general population. The genes and pathways identified from these analyses indicate roles for calcium signaling, myocyte internal structure and interconnections as well as autonomic control as key mechanisms (10,11). Combined, these loci explain ~10% of the trait variability in studies using common SNVs, suggesting more undiscovered loci are likely to be involved (10). Furthermore, it is recognized that women have longer intervals compared with men (12), which may suggest sexually dimorphic genetic effects, but at present no systematic searches have been conducted.

The aim of this work was to (i) provide new candidate genes, (ii) identify pleiotropic associations with other cardiovascular traits and (iii) scan for sexually dimorphic genetic effects.

**Figure 1:**
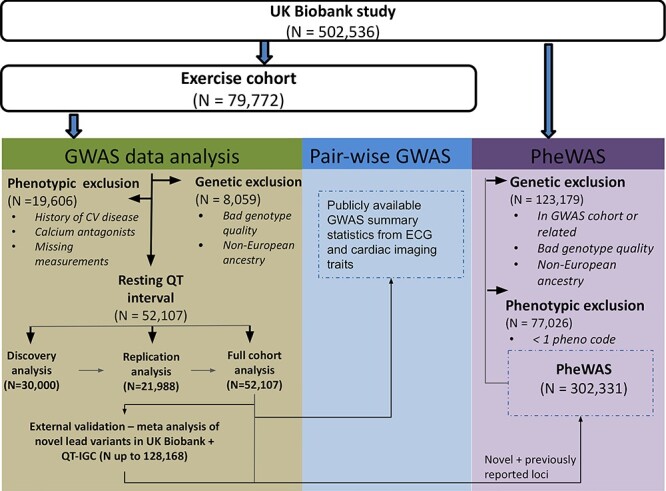
Overview of the study design. From the full UK Biobank cohort, we selected all individuals from the exercise cohort who had measured their QT interval at resting period (pre-exercise) and applied genotypic and phenotypic quality control. Three separate GWASes were conducted: discovery, replication and a combined full-cohort analysis. First- and second-degree–related individuals to the discovery cohort were not included in the replication cohort. Novel lead variants were externally validated in a meta-analysis.

## Results

### Identification of novel loci

**Figure 2:**
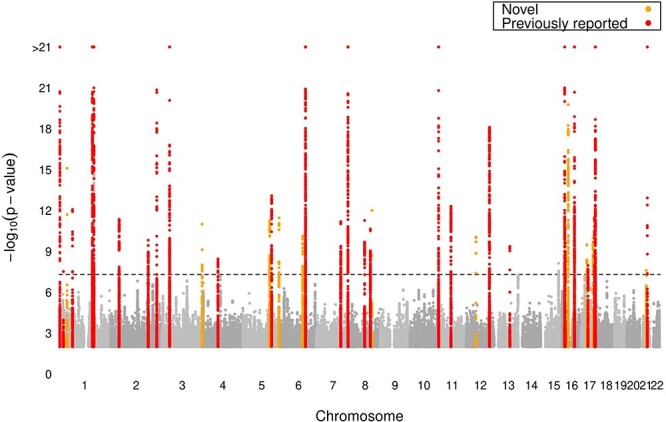
Manhattan plot showing known (red) and novel (orange) loci associated with resting QT interval in the full dataset. Eleven novel loci were discovered by testing ~9.8 million genetic variants (each represented by a dot) in 52 107 individuals from UK Biobank. The *x*-axis represents the genome in physical order, and the *y*-axis represents *P*-values (−log10(*P*-value)) of association. The black horizontal dotted line represents the genome-wide significance threshold (*P* < 5 × 10–8). One potentially novel locus did not pass external validation: (*ZNF592*, *rs8023658, chr15:85323220*).

We tested association between ~9.8 million genetic variants (minor allele frequency (MAF) > 0.01) and resting QT interval in 52 107 individuals of European ancestry from UK Biobank. An overview of the study design is presented in Figure 1. We randomly divided our dataset into a discovery (*N* ~ 30 000) and replication (*N* ~ 22 000) cohort (Supplementary Material, Table S1). In the discovery phase, 21 loci (considering one lead SNV per 1 Mb region) reached genome-wide significance (*P* < 5 × 10^−8^). There were variants at 14 potential novel loci with suggestive significance (*P* < 1 × 10^−6^) (Supplementary Material, Table S2), and these were taken forward for replication in ~22 000 independent samples from UK Biobank. Eight out of fourteen novel SNVs formally replicated (*P* ≤ 0.05/14 = 0.0036) and all had concordant directions of effect (Table 1, Supplementary Material, Table S3). Given the limited sample size of the discovery cohort, we also performed a full dataset GWAS (Fig. 1). In total, 39 loci were genome-wide significant and 12 were potentially novel. The QQ plots for the discovery phase and full dataset GWAS (Supplementary Material, Fig. S2) did not show evidence of population stratification or inflation. To seek external validation of the 12 potentially novel loci, we performed a meta-analysis of results from the QT Interval-International GWAS Consortium (QT-IGC) summary statistics (*N* up to ~76 000) (10). Four lead variants and their proxies were not available for lookup (*KCNQ4*, *ZFPM2*, *ARID2* and *NUFIP2*). For the remaining eight loci, seven were genome-wide significant (*P* < 5 × 10^−8^) with concordant direction of effects (*TMEM44*, *SLC27A6*, *NKX2–5*, *PREP*, *ATP2A1*, *YPEL2* and *LINC00189*). In total, we report 11 novel loci, of which 7 were externally validated (Table 1, Fig. 2, Supplementary Material, Table S4). Regional plots of the novel loci are shown in Supplementary Material, Figure S3A.

To identify independent variants, approximate stepwise conditional analysis using genome-wide complex trait analysis (GCTA) was performed (Materials and Methods). Six secondary variants were identified as being independent of the lead signal (*r*^2^ < 0.1) (Table 1, Supplementary Material, Table S5, Supplementary Material, Fig. S4). One independent signal was found at the novel *PREP* locus (rs144483936, Supplementary Material, Fig. S4A), approximately 126 Kb upstream of the lead SNV at this locus (rs2793409). The heritability of resting QT interval using the total genome-wide genetic variation captured in this study was 28.9% (h2g SNV heritability). The combination of 11 novel SNVs improved the % variance explained of QT from ~10% (obtained when considering previously reported SNVs only) to 11%.

We also performed a lookup of previously reported variants for QT interval. The lead variant or proxy was available for 47/48 loci; 42 (88%) had at least one variant with a nominal significant association (*P* < 0.05) in the full cohort, and all but one had concordant direction of effects. Thirty-four loci (73%) passed the formal replication Bonferroni threshold (*P* < 0.001 (=0.05/47; Supplementary Material, Table S6), all with concordant directions of effect. Of the remaining five loci without significant association, the direction of effect was concordant for two loci.

### Sex-stratified analyses

A sex-stratified GWAS in the full cohort including 24 495 males and 27 612 females identified two novel loci meeting the genome-wide significance threshold (*P* < 5 × 10^−8^, Table 1, Supplementary Material, Table S7, Supplementary Material, Fig. S3B and C). The *RASGRF2* locus with index variant rs10063881 was significantly associated with resting QT interval in men (*P* = 3.4 × 10^−8^), but not in women (*P* = 6.2 × 10^−1^). Conversely, the *PKP2* locus with index variant rs11052242 showed a significant association in women (*P* = 2.5 × 10^−9^), but not in men (*P* = 6.8 × 10^−1^). Neither locus was genome-wide significant in the full cohort. The effect estimates at the two sex-specific loci were significantly different across both sexes (*P* = 2.1 × 10^−4^ and *P* = 7.9 × 10^−5^ for *RASGRF2* and *PKP2,* respectively, Supplementary Material, Table S7).

### Bioinformatics: key pathways, variant annotation and candidate genes

**Figure 3:**
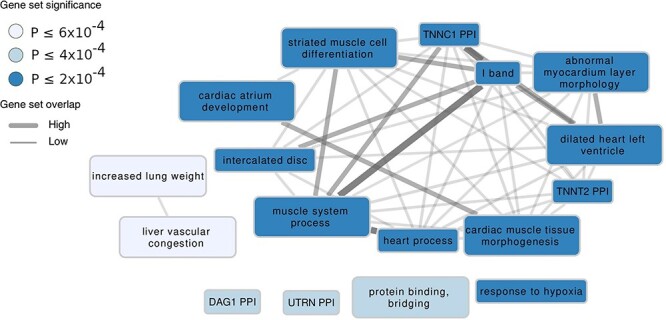
Reconstituted gene sets implicated in resting QT interval. Clustered reconstituted gene sets (out of 93 with FDR < 0.05) found by DEPICT to be significantly enriched for genes in QT-associated loci. Each node, colored according to the permutation *P*-value, represents a gene set and the gray connecting lines represent pairwise overlap of genes within the gene sets. Abbreviations: PPI: protein–protein interaction, DAG1: Dystroglycan 1, TNNC1: Troponin C1, UTRN: Utrophin, TNNT2: Troponin T2.

We performed Data-driven Expression Prioritized Integration for Complex Traits (DEPICT) (13) analysis including all genome-wide significant SNVs (*P* < 5 × 10^−8^) in the full dataset GWAS summary statistics to detect gene sets that are enriched for genes at QT interval loci. Of the 14 461 gene sets tested, 93 were enriched (false discovery rate (FDR) < 5%). To decrease redundancy, we clustered the enriched gene sets based on pairwise Pearson correlation (Materials and Methods) into 17 gene set clusters (Fig. 3, Supplementary Material, Table S8). Pathways and biological processes in which these gene sets are enriched included those involved in cardiomyocyte contraction, cardiac atrial development and structural remodeling of the myocardium. DEPICT also detected significant enrichment in 10 tissue types (FDR < 0.05). The top four tissues (FDR < 0.01) were all cardiac:heart atria (*P* = 9.83 × 10^−6^), heart (*P* = 1.24 × 10^−5^), atrial appendage (*P* = 1.72 × 10^−5^) and heart ventricles (*P* = 1.82 × 10^−5^) (Supplementary Material, Table S9). In addition, we also found significant enrichment in gastrointestinal and musculoskeletal tissues.

**Table 1:** Loci associated with resting QT interval.

						Discovery				Replication				Combined			
Locus	SNV	CHR	BP	EA	EAF	*P*	*N*	*β*	SE	*P*	*N*	*β*	SE	*P*	*N*	*β*	SE
*Novel*																	
**KCNQ4**	**rs116015634**	**1**	**41 250 961**	**C**	**0.98**	**5.00E−10**	**29 596**	**0.112**	**0.018**	**5.60E−07**	**21 988**	**0.11**	**0.02**	**7.90E−16**	**52 107**	**0.11**	**0.01**
**TMEM44** ^a^	**rs1706003**	**3**	**194 299 967**	**G**	**0.53**	**8.50E−08**	**28 572**	**0.030**	**0.006**	**2.40E−05**	**21 227**	**0.03**	**0.01**	**1.00E−11**	**50 305**	**0.03**	**0.00**
*RASGRF2* ^b^	rs10063881	5	80 278 715	A	0.91	**–**	**–**	**–**	**–**	**–**	**–**	**–**	**–**	3.40E**−**08	24 126	0.059	0.011
**SLC27A6** ^a^	**5:128147544_CCTTCCTTCCTTT_C**	**5**	**128 147 544**	**CCTTCCTTCCTTT**	**0.56**	**8.60E−08**	**27 958**	**0.030**	**0.006**	**4.70E−05**	**20 771**	**0.027**	**0.007**	**2.90E−11**	**49 223**	**0.028**	**0.004**
**NKX2–5** ^a^	**rs35564079**	**5**	**172 670 611**	**C**	**0.72**	**3.00E−07**	**28 852**	**−0.031**	**0.006**	**3.90E−06**	**21 435**	**−0.033**	**0.007**	**3.50E−12**	**50 797**	**−0.032**	**0.005**
PREP^a,c^	rs2793409	6	105 710 719	G	0.87	1.40E**−**04	29 471	0.031	0.008	4.10E**−**08	21 895	0.052	0.009	7.50E**−**11	51 887	0.039	0.006
**ZFPM2**	**rs72671655**	**8**	**106 347 897**	**T**	**0.96**	**2.80E−08**	**29 596**	**−0.079**	**0.014**	**3.40E−05**	**21 988**	**−0.069**	**0.017**	**1.00E−12**	**52 107**	**−0.076**	**0.011**
*PKP2* ^d^	rs11052242	12	32 950 367	G	0.87	**–**	**–**	**–**	**–**	**–**	**–**	**–**	**–**	2.50E**−**09	27 402	-0.050	0.008
ARID2	rs78341918	12	46 199 798	T	0.96	1.70E**−**05	29 236	0.064	0.015	8.40E**−**06	21 721	0.077	0.017	8.70E**−**11	51 474	0.072	0.011
**ATP2A1** ^a^	**rs9933198**	**16**	**28 888 409**	**C**	**0.76**	**5.80E−10**	**29 596**	**0.039**	**0.006**	**8.00E−07**	**21 988**	**0.037**	**0.008**	**8.40E−16**	**52 107**	**0.038**	**0.005**
**NUFIP2**	**rs56336338**	**17**	**27 645 258**	**G**	**0.86**	**1.60E−07**	**29 065**	**−0.041**	**0.008**	**1.20E−03**	**21 594**	**−0.029**	**0.009**	**3.50E−10**	**51 172**	**−0.037**	**0.006**
**YPEL2** ^a^	**rs142166837**	**17**	**57 471 022**	**C**	**0.52**	**4.50E−07**	**29 222**	**−0.028**	**0.005**	**7.00E−04**	**21 710**	**−0.022**	**0.006**	**1.00E−10**	**51 448**	**−0.027**	**0.004**
LINC00189^a^	rs2832274	21	30 600 189	A	0.60	1.10E**−**06	29 288	−0.027	0.006	3.40E**−**03	21 759	−0.019	0.007	2.50E**−**08	51 564	-0.023	0.004
*Previously reported*																	
RNF207	rs846111	1	6 279 370	G	0.73	4.90E**−**24	29 596	−0.062	0.006	3.00E**−**18	21 988	−0.062	0.007	6.30E**−**41	52 107	-0.061	0.005
TCEA3	rs2298632	1	23 710 475	C	0.50	1.40E**−**04	28 452	−0.021	0.006	7.80E**−**04	21 138	−0.022	0.006	3.00E**−**08	50 092	-0.023	0.004
SGIP1	rs10789207	1	66 991 346	T	0.78	4.30E**−**10	29 355	−0.042	0.007	8.60E**−**04	21 809	−0.026	0.008	8.50E**−**13	51 682	-0.035	0.005
NOS1AP	rs12143842	1	162 033 890	C	0.75	2.20E**−**104	29 596	−0.136	0.006	2.70E**−**73	21 988	−0.133	0.007	2.10E**−**180	52 107	-0.135	0.005
DPT^c^	rs533619	1	168 685 805	C	0.73	4.10E**−**16	29 575	−0.050	0.006	4.40E**−**17	21 973	−0.060	0.007	1.40E**−**32	52 070	-0.054	0.005
LOC101929667	rs17026114	2	40 743 183	A	0.96	2.30E**−**09	29 308	0.081	0.013	2.70E**−**04	21 774	0.057	0.016	4.50E**−**12	51 599	0.070	0.010
TTN^c^	rs12993099	2	179 598 228	A	0.92	1.80E**−**06	29 357	0.049	0.010	1.30E**−**05	21 810	0.052	0.012	1.50E**−**10	51 686	0.049	0.008
SLC4A3	rs35394392	2	220 500 830	G	0.78	4.60E**−**14	29 478	−0.050	0.007	5.20E**−**09	21 900	−0.045	0.008	1.40E**−**21	51 899	-0.047	0.005
SCN5A-SCN10A	rs76521806	3	38 699 265	A	0.02	1.50E**−**14	27 447	0.154	0.020	7.80E**−**11	20 391	0.156	0.024	5.10E**−**26	48 323	0.160	0.015
SLC4A4	rs5859257	4	72 115 860	T	0.14	1.70E**−**06	29 093	0.038	0.008	4.60E**−**04	21 614	0.033	0.009	3.60E**−**09	51 221	0.035	0.006
RNU6-1148P	rs542777476	5	137 406 243	C	0.17	2.40E**−**10	29 593	0.045	0.007	1.50E**−**04	21 986	0.032	0.008	8.40E**−**14	52 102	0.040	0.005
SLC35F1	rs11153730	6	118 667 522	T	0.51	1.20E**−**28	29 596	−0.061	0.005	1.10E**−**23	21 988	−0.064	0.006	4.90E**−**53	52 107	-0.063	0.004
CAV1	rs1997571	7	116 198 621	G	0.41	2.40E**−**07	29 514	0.029	0.006	2.10E**−**06	21 927	0.031	0.007	6.60E**−**12	51 962	0.029	0.004
KCNH2	rs1805120	7	150 649 531	G	0.79	5.90E**−**25	29 360	−0.069	0.007	2.80E**−**23	21 813	−0.077	0.008	5.40E**−**48	51 691	-0.073	0.005
NCOA2	rs4738080	8	71 129 813	A	0.07	1.90E**−**07	29 511	0.056	0.011	1.20E**−**04	21 925	0.049	0.013	5.40E**−**12	51 957	0.056	0.008
LAPTM4B	rs11777388	8	98 808 872	A	0.59	4.60E**−**08	28 676	0.031	0.006	2.80E**−**04	21 305	0.024	0.007	2.30E**−**11	50 487	0.028	0.004
KCNQ1	rs2074238	11	2 484 803	T	0.09	3.90E**−**103	29 596	−0.205	0.009	2.80E**−**59	21 988	−0.185	0.011	1.20E**−**164	52 107	-0.197	0.007
TMEM258	rs102275	11	61 557 803	T	0.65	3.60E**−**09	29 596	0.034	0.006	8.00E**−**05	21 988	0.026	0.007	4.90E**−**13	52 107	0.031	0.004
ATP2A2	rs3026482	12	110 780 540	A	0.77	3.30E**−**11	29 506	−0.043	0.007	1.40E**−**08	21 921	−0.043	0.008	7.80E**−**19	51 948	-0.043	0.005
KLF12	rs1886512	13	74 520 186	T	0.63	2.60E**−**07	29 537	−0.029	0.006	3.70E**−**04	21 944	−0.024	0.007	4.40E**−**10	52 004	-0.027	0.004
LITAF	rs7187498	16	11 687 879	A	0.54	7.20E**−**15	29 497	0.043	0.005	7.30E**−**15	21 915	0.050	0.006	9.30E**−**29	51 933	0.046	0.004
MIR193BHG	rs30224	16	14 406 119	C	0.65	4.20E**−**07	29 024	−0.029	0.006	5.60E**−**05	21 563	−0.027	0.007	2.30E**−**11	51 099	-0.029	0.004
NDRG4	rs150728296	16	58 547 511	C	0.75	2.50E**−**25	29 460	0.066	0.006	8.40E**−**21	21 887	0.069	0.007	4.20E**−**47	51 867	0.069	0.005
RAD51L3-RFFL	rs1680529	17	33 405 262	C	0.44	4.50E**−**06	29 526	0.025	0.005	8.60E**−**04	21 936	0.021	0.006	1.10E**−**08	51 985	0.024	0.004
PRKCA	rs9909004	17	64 306 133	C	0.42	3.50E**−**06	29 503	−0.026	0.006	4.10E**−**08	21 919	−0.036	0.007	3.30E**−**12	51 943	-0.029	0.004
KCNJ2	rs4399570	17	68 479 345	G	0.70	2.70E**−**18	29 480	0.052	0.006	4.20E**−**10	21 902	0.044	0.007	6.50E**−**28	51 902	0.049	0.004
KCNE1	rs1805128	21	35 821 680	C	0.99	1.70E**−**21	29 596	−0.224	0.024	1.60E**−**16	21 988	−0.227	0.028	1.70E**−**38	52 107	-0.229	0.018

Results are reported for the discovery (*N* = 30 000), independent replication (*N* = 22 000) and combined (*N* = 52 000) cohorts. The locus name indicates the gene that is in the closest proximity to the most associated SNV. Replicated SNVs in UK Biobank are indicated in bold type. Seven novel loci passed external validation in the QT Interval-International GWAS Consortium (marked with superscript a), four loci were not available for lookup (*KCNQ4*, *ZFPM2*, *ARID2* and *NUFIP2*) and one locus did not pass external validation: (*ZNF592,* rs8023658). Two remaining loci were sex-specific (*RASGRF2* and *PKP2*). SNV: single-nucleotide variant, CHR: chromosome, BP: base pair position, based on HG build 19, EA: effect allele, EAF: effect allele frequency from discovery dataset, *β*: beta in beats per minute, SE: standard error, *N*: effective number of participants, *P*: *P*-value.

^b^Sex-specific locus: association was only significant in males.

^c^Secondary SNVs identified at same locus using conditional analysis: rs28362572, 1:169094566_CTTACATCCG_C and rs72706975 at *DPT* locus; rs141324241 and 2:179768491_TA_T at *TTN* locus; rs144483936 at *PREP* locus.

^d^Sex-specific locus: association was only significant in females.

Using Functional element Overlap analysis of the Results of GWAS Experiments (FORGE), we identified significant (FDR < 0.05) cell type–specific enrichment within DNase I–hypersensitive sites that are transcriptionally active in fetal heart samples (Supplementary Material, Fig. S5). These data suggest that resting QT variants are involved in transcriptional regulation in the development of the fetal heart.

We observed non-synonymous variants only at four previously reported loci. At two loci, these were the index variant (Supplementary Material, Table S10). As the majority of resting QT variants are non-coding, we also identified regulatory variants, which might affect gene expression levels of their target genes. We interrogated publicly available expression quantitative trait loci (eQTL) datasets of heart, adrenal and brain tissues through Genotype-Tissue Expression (GTEx) (14) to highlight potential genes influencing the resting QT interval through expression at each of the discovered loci, including novel and previously reported loci (Materials and Methods). In total, 10 different transcripts colocalized with 9 loci for resting QT interval (Supplementary Material, Table S9). Three of these transcripts colocalized with genes at novel loci. *NKX2–5* (brain cerebellum) and *YPEL2* (heart left ventricle) and the index variant rs1706003 near *TMEM44* colocalized with expression levels of *ATP13A3* in both heart atrial appendage and left ventricle tissues (Supplementary Material, Table S11). The remaining seven transcripts colocalized with six previously reported loci for QT interval. The locus with lead variant rs12143842 near *NOS1AP* colocalized with expression levels of both *C1orf226* (heart left ventricle and atrial appendage) and *NOS1AP* (heart left ventricle). At the remaining five loci, we found one colocalization per locus with the expression levels from the nearest gene: *TCEA3* (heart left ventricle)*, SGIP1* (heart left ventricle), *LITAF* (heart left ventricle), *RFFL* (heart left ventricle) and *PRKCA* (heart left ventricle, Supplementary Material, Table S9).

Genetic variants may also have a causal effect through long-range target genes of non-coding variants because of regulatory chromatin interactions. To identify distal candidate genes, we explored chromatin interaction (promotor capture Hi-C) data for all lead and secondary variants with regulatory potential. We identified several genes whose promoter regions form significant chromatin interactions in brain, heart and adrenal tissue at 9 novel and 17 previously reported loci (Supplementary Material, Table S12). An overview of all candidate genes per locus is provided in Supplementary Material, Table S13 (novel loci) and Supplementary Material, Table S14 (previously reported loci). The bioinformatics analyses, coupled with literature review and information on knock-out mouse models were used to select ‘final’ candidate gene(s) at a locus. If no gene had strong support, we indicate the nearest gene as the ‘final’ candidate gene.

**Figure 4:**
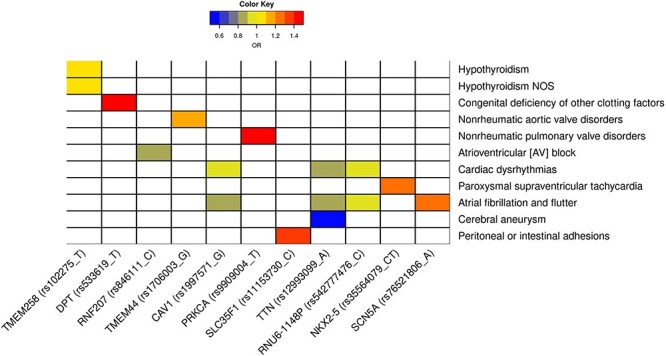
PheWAS results for resting QT interval variants. All primary and secondary variants discovered for resting QT interval were tested in ~302 000 individuals for 1238 phenotypes. Significant associations were found for the 11 variants displayed. The color corresponds to the risk estimate [odd ratio (OR)] for each phenotype with respect to the QT interval prolonging risk allele, where blue indicates a reduced risk and red an increased risk.

**Figure 5:**
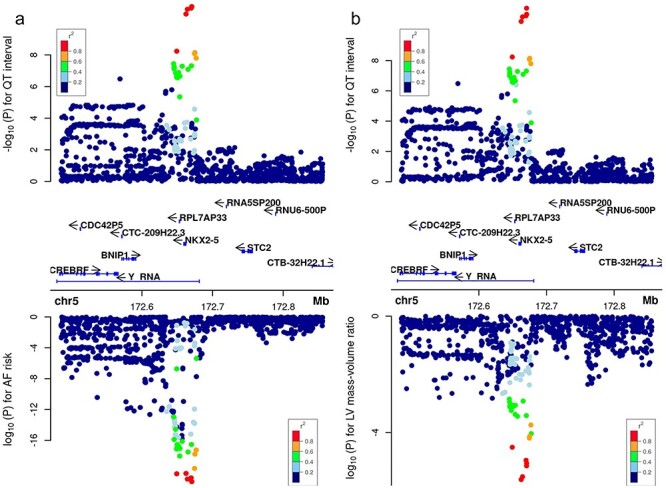
Cardiac pleiotropy near NKX2–5. Shared genetic influences between QT interval and atrial fibrillation risk (**A**) and imaging-derived LV mass–volume ratio (**B**).

### Pleiotropy of genetic variants associated with resting QT interval

To explore shared mechanisms of disease, we first queried PhenoScanner (13) and National Human Genome Research Institute–European Bioinformatics Institute (NHGRI-EBI) GWAS Catalog (14) for genome-wide phenotype–genotype association of all novel lead and secondary variants (including two sex-specific loci) and their proxies (r^2^ > 0.8). Trait associations were found for five loci: the SNV at *TMEM44* was associated with diastolic blood pressure, *SLC27A6* with red cell distribution, *NKX2–5* with heart rate and atrial fibrillation, and finally *NUFIP2* with mean platelet volume (Supplementary Material, Table S15). To facilitate the identification of novel associations between QT interval variants and other phenotypes, we also performed a phenome-wide association study on 302 331 unrelated individuals from UK Biobank not included in the GWAS (Supplementary Material, Fig. S1). In this analysis, we tested for associations between the 44 index and secondary SNVs discovered and 1238 phenotypes derived from the hospital episodes statistics (Methods). Significant associations were found for 11 index variants, of which 6 were associated with atrial pathology including atrioventricular block, atrial fibrillation or paroxysmal supraventricular tachycardia (Fig. 4). Interestingly, QT-prolonging alleles were associated with a decreased risk for four of these traits. The existence of shared genetic influences between resting QT interval and atrial and ventricular phenotypes was further assessed using a Bayesian bivariate analysis as implemented in GWAS-PW (15) using GWAS summary statistics of ECG markers (PR, QRS, Tpeak-Tend interval, QT interval dynamics during exercise and atrial fibrillation) (16–19) and several left ventricular–derived imaging phenotypes (20,21). Substantial overlap was observed with other ECG markers of ventricular depolarization and repolarization: from the 38 resting QT interval loci discovered in the main analysis, 27 (71%), 30 (79%) and 22 (58%) loci shared a genetic effect (posterior probability >90%) with QRS duration, QT dynamics and Tpeak-Tend, respectively. Shared genetic effects were also observed at 15 (39%) and 12 (32%) of the QT loci for atrial fibrillation risk and PR interval, respectively, but with no consistent effect for direction. Finally, three loci (8%) overlapped with imaging-derived phenotypes from the heart (Supplementary Material, Table S16). Figure 5 shows examples of the shared genetic influence at *NKX2–5* between resting QT interval and atrial fibrillation (A) and left ventricular mass–volume ratio (B).

## Discussion

Using a robust framework of independent discovery and validation samples, we discovered 13 novel loci (including two sex-specific loci) for resting QT interval. Bioinformatics and pleiotropic analyses highlighted new pathways in regulating QT interval related to myocardial structure and implicated genes important in atrial pathology.

### Implication in myocardial structure

Gene set enrichment of QT interval loci highlights novel candidate genes involved in myocardial structure and atrial phenotypes including atrium morphogenesis and development and abnormal heart atrium morphology. DEPICT identified significant enrichment for hypertrophic cardiomyopathy phenotypes with support from Kyoto Encyclopedia of Genes and Genomes (KEGG) and mouse phenotypes. Within this gene set is novel candidate gene *NKX2–5,* a cardiac homeobox transcription factor expressed in a broad range of cardiac sublineages (22). Multiple lines of evidence document that the loss of *NKX2-5*-derived cues leads to defects in the morphogenesis, maturation and specification of the AV nodal and working myocyte cell lineages (22,23). Clinical manifestations include dilated cardiomyopathy and sudden cardiac death (24), possibly due to disarray of myocytes affecting both depolarization and repolarization (25). Interestingly, we observed colocalization of the index variant with expression levels of *NKX2-5* in brain cerebellum, but not in cardiac tissues, although pairwise GWAS analysis results did indicate shared genetic effects with cardiac phenotypes derived from cardiac imaging. Although the exact mechanisms of N*KX2-5* in regulating the QT interval remain unclear, we observed shared genetic influences with ECG QRS duration, Tpeak-Tend interval and imaging-derived left ventricular phenotypes (Fig. 5, Supplementary Material, Table S16). We did not find significant overlap with PR interval, but a recent study (16) reported a secondary variant at this locus (rs954635, lead variant = rs29795), which is a close proxy (*r*^2^ = 0.82) of the lead variant for QT interval in our analysis. These observations indicate significant pleiotropic effects at *NKX2–5* between the QT interval and other ECG traits.

### Genetic factors underlying resting QT interval are implicated in atrial pathology

Our PheWAS analysis showed that several QT interval variants exhibit pleiotropic effects with supraventricular arrhythmias, including atrial fibrillation. In addition, pairwise GWAS analyses showed that approximately a third of the resting QT interval loci are shared with the ECG PR interval and atrial fibrillation risk. These results suggest potentially important overlap in the genetic architecture between resting QT interval and atrial pathologies. Indeed, it is well recognized that individuals with a prolonged or shortened QT interval are at increased risk of atrial fibrillation (26,27). The exact mechanisms underlying this association remain to be investigated, but abnormal atrial electrical activity is a risk factor for atrial fibrillation and the corresponding ion channel complexes involved are similar in atrial and ventricular tissues (28), which may explain shared biology between atrial fibrillation and ventricular repolarization. Our results from the pairwise GWAS support this hypothesis, suggesting shared genetic effects between QT interval and atrial fibrillation risk (Supplementary Material, Table S16).

### Sex-specific loci for resting QT interval

Preliminary exploration of sexually dimorphic genetic effects of resting QT interval suggested two sex-specific loci. The locus at *PKP2* was only associated with resting QT interval in women. A candidate at this locus, *PKP2* encodes Plakophilin 2, which plays an important role in the gap junction formation and maintenance at cell–cell contact sites in the ventricular myocardium (29). Mutations in *PKP2* are an important cause of arrhythmogenic ventricular cardiomyopathy, which is characterized by loss of cell–cell communication disrupting gap junction coupling and Na^+^channel trafficking affecting the activation of the myocardium (29). In males, we identified a sex-specific locus near Ras protein-specific guanine nucleotide-releasing factor 2 (*RASGRF2*). This protein helps to regulate neuronal excitability, neuronal survival in response to ischemia, learning and memory formation (30,31). It may be possible that this protein affects QT interval by modulating autonomic cardiac function (32). Future studies with larger sample sizes may permit replication of these findings and allow more detailed investigation of the genetic basis of sex-specific QT interval values.

Our study has some limitations. Six loci require formal replication in an independent dataset, and this includes the four loci that were not available for lookup in the QT-IGC GWAS and two sex-specific loci. Furthermore, our results are limited to individuals from European ancestry. Finally, validation will be required for the results from the PheWAS, where characteristics of the validation cohort are similar to the cohort described here (33).

In summary, we identified 13 novel loci for resting QT interval. Bioinformatics analyses implicated several new candidate genes for future functional studies. By studying the shared biological mechanisms between resting QT interval and atrial pathology, we further enhance our understanding of the mechanisms modulating the QT interval. Finally, preliminary analysis suggests there may be some sex-specific loci for QT interval.

## Materials and Methods

### UK Biobank

Phenotypic and genetic data were obtained from the UK Biobank. UK Biobank is a prospective study of ~500 000 volunteers, comprising even numbers of men and women aged 40–69 years on recruitment, with extensive baseline and follow-up clinical, biochemical, genetic and outcome measures (34). The UK Biobank study has approval from the North West Multi-Centre Research Ethics Committee, and all participants provided informed consent. Genotyping was performed centrally by UK Biobank using the Applied Biosystems UK BiLEVE Axiom Array or the UK Biobank AxiomTM Array and SNVs were imputed using the Haplotype Reference Consortium (HRC) and the 1000 genomes (1000G) reference panels (34). Data used in this study were part of UK Biobank application number 8256 and anonymized data and materials generated in this work have been returned to UK Biobank and can be accessed per request.

### Quality control

Genetic quality control (QC) was performed on the set of individuals who were invited to an exercise stress test ECG recording (*N* = 79 772, Fig. 1). Individuals with bad genotype quality, provided by UK Biobank, i.e. high missingness or heterozygosity and discordance between the self-reported sex and the sex inferred from the genotypes were excluded (*N* = 2655). We then restricted our dataset to individuals of European ancestry by applying k-means clustering to the principal component analysis (PCA) data resulting in *N* = 71 713 individuals for phenotypic QC (Supplementary Material, Supplemental Methods). We then applied phenotypic quality control and excluded individuals with a known history of cardiovascular disease, including atrial fibrillation, history of myocardial infarction or heart failure, (supra)-ventricular tachycardia, atrioventricular nodal re-entrant tachycardia, second- or third-degree atrioventricular block, bundle branch block and use of a pacemaker. Furthermore, we excluded individuals on heart rate–altering medications (non-dihydropyridine calcium antagonists (Anatomic Therapeutic Chemical (ATC) code C08D, digoxin (ATC code C01AA5) and amiodarone (ATC code C01BD01)) as these could affect QT interval. We also excluded individuals with an extreme heart rate during (<40 or >120 bpm) and individuals with poor-quality ECG recording by manually reviewing ECGs with a low signal-to-noise ratio, resulting in 52 107 individuals for analysis.

### Phenotypic data

The exercise stress test included an initial 15 s phase at rest, which was used in this study to estimate the average resting QT for each individual. Details on ECG pre-processing have been published previously (17,18). Briefly, we constructed an averaged ECG waveform by aligning and averaging all heart beats within the 15 s window and then measuring automatically the interval between the QRS onset and end of the T-wave using the tangent method (35).

### Genetic analyses and percent variance explained

Inverse-normal transformation was applied before genetic analyses corrected for skewed distributions of the resting QT interval (Supplementary Material, Fig. S6). GWASs were performed to discover SNVs associated with resting QT interval using a linear mixed model method (BOLT-LMM, Supplementary Material, Supplemental Methods) (36). We included sex, diabetes, age, body mass index, genotyping array (binary indicator: UK Biobank versus UK BiLEVE) and resting RR interval as covariates. Since we did not have access to an independent study that could serve as a replication study, we randomly divided our dataset into discovery (*N* ~ 30 000) and replication (*N* ~ 22 000) datasets and removed related individuals (kinship coefficient > 0.088) (34). We also performed a GWAS in the full dataset, as well as sex-stratified GWASs (27 612 females, 24 495 males) to identify additional loci. Differences between men-specific and women-specific beta estimates were tested for significance using the t statistic (Supplementary Material, Supplemental Methods) (37). To compute the percent of variance explained, we first generated the residuals from the regression of the trait against the covariates included in the GWAS analysis. We then fit a second linear model for the trait residuals with all the sentinel variants and the first 10 principal components.

### Conditional analyses

We examined the existence of SNVs independent to lead SNVs but tagging the same loci by applying genome-wide complex trait analysis (GCTA) (45) for all validated and genome-wide significant loci from the full dataset GWAS. We used a stringent approach to declare a secondary signal based on three criteria: (i) the newly identified SNV original *P-*value was lower than 1 × 10^−6^; (ii) there was less than a 1.5-fold difference between the lead SNP and secondary association *P-*values on a −log_10_ scale, i.e. if −log_10_(*P*_lead_)/−log_10_(*P*_sec_) < 1.5; and (iii) if there was less than a 1.5-fold difference between the main association and conditional association *P-*values on a −log_10_ scale, i.e. if −log_10_(*P*_sec_)/−log_10_(*P*_cond_) < 1.5. The 1.5-fold threshold was chosen to offer a good compromise between reducing the risk of picking up false positives and remaining sensitive to detect true secondary signals (38,39).

### Criteria for claiming novel loci

We defined loci based on a 1 MB (±500 kb) window around each sentinel variant. To determine whether a discovered locus was novel or known, we downloaded all reported SNVs for resting QT from the PhenoScanner (13) and NHGRI GWAS catalog (14) (URLs). At the time of writing, the catalogues had not been updated with the latest results from Bihlmeyer *et al*. (11), who identified additional six and three novel loci, respectively, which we added to the list. Variants were filtered for genome- or exome-wide significance (*P* = 5 × 10^−8^ or *P* = 2 × 10^−7^, respectively). We then calculated the pairwise linkage disequilibrium (LD) within a 4 Mb region (±2 Mb) around each variant using PLINK v2 (40). Next, variants in LD (*r*^2^ > 0.1) were ordered according to their positions on the chromosome and we defined a window with the start position of the first variant and the end position of the last variant. The start and end of the window was extended by 50 kb on either side. This LD defined window or a window of ±500 kb, which ever was the larger, was considered as the previously reported locus. If the sentinel SNV or its proxy (*r*^2^ > 0.8) was not in UK Biobank, then a locus was defined by window of ±500 kb around the variant using its chromosome and base pair position.

### Bioinformatics analyses

We performed comprehensive bioinformatics analyses to annotate loci at both variant (all SNVs in linkage disequilibrium (LD), *r*^2^ ≥ 0.8, were considered) and gene level. For each lead SNV, we annotated the nearest genes and genes in which SNVs in LD (*r*^2^ > 0.4) with the lead SNV are located using University of California, Santa Cruz (UCSC) known genes. Variant effect predictor (VEP) analyses determined the effect of the variants, including the impact of amino acid substitutions (41). We also investigated whether any of the discovered GWAS signals co-localized with genetic variants that regulate expression in adrenal, heart and brain tissues using expression quantitative trait locus (eQTL) signals from the Genotype-Tissue Expression (GTEx) dataset version 7 (42) using COLOC R-package (43). We analyzed the following eQTL tissues as they were hypothesized to potentially host key biological mechanisms in regulating QT interval, including autonomic nervous activity (44): brain amygdala, brain anterior cingulate cortex BA24, brain caudate basal ganglia, brain cerebellar hemisphere, brain cerebellum, brain cortex, brain frontal cortex BA9, brain hippocampus, brain hypothalamus, brain nucleus accumbens basal ganglia, Brain putamen basal ganglia, brain spinal cord cervical c-1, brain substantia nigra, heart atrial appendage, heart left ventricle and adrenal gland. Potential target genes of regulatory SNVs in high LD (*r*^2^ ≥ 0.8) with lead and secondary variants were identified using long-range chromatin interaction (Hi–C) data (45). In addition, DEPICT analyses prioritized likely causal genes and tissues (46). Enrichments with FDR < 0.05 were deemed significant. The Affinity Propagation tool (47) was used for clustering based on pairwise Pearson correlation between significant gene sets and clusters were named by their ‘representative’ gene set, which was automatically chosen by the clustering method. The gene set enrichment analysis was visualized in Cytoscape (48). A literature review examined all identified genes at a locus. We also queried gene-specific animal models using International Mouse Phenotyping Consortium and the Mouse Genome Informatics database (49). The bioinformatics analysis, coupled with literature review and information on knock-out mouse models, was used to select ‘final’ candidate gene(s) at a locus. If no gene had strong support, we indicate the nearest gene as the ‘final’ candidate gene.

We performed PheWAS analysis in 310 000 unrelated European ancestry individuals from UK Biobank who were not included in the GWAS. Ancestry definition and sample QC exclusions were performed in the same manner as for the GWAS. Next, we extracted all ICD-9 and ICD-10 codes from the hospital episode statistics of the included individuals and mapped them to phecodes using the latest phecode definitions downloaded from PheWAS resources (URLs) (50). We then run a PheWAS for the 47 lead and secondary variants discovered for resting QT interval using the PheWAS package in R (51) with covariate adjustment for sex. We also investigated whether loci associated with resting QT interval exert a shared effect on other ECG and cardiac imaging traits. We downloaded GWAS summary statistics for ECG conduction and repolarization traits (PR-interval, Tpe interval and QT dynamics) (16–18), atrial fibrillation (19) and cardiac imaging traits (20,21), including left ventricular ejection fraction, mass, end systolic volume, end diastolic volume, mass–volume ratio and trabecular morphology. We also analyzed the overlap with ECG QRS duration. As we had no access to suitable summary statistics, we first performed a GWAS for QRS duration in ~51 000 QCed samples from the same UK Biobank cohort in which we have analyzed QT interval. We adjusted for the following covariates: sex, age, body mass index, height and genotyping array. The GWAS was performed using BOLT-LMM software (36), similar to QT interval.

The genome was divided into approximately independent blocks based on previously reported coordinates identified using patterns of linkage disequilibrium in the European populations in the 1000 Genomes Project (52). For each trait, we then performed pairwise GWAS (15) to scan for overlapping association signals with a posterior probability greater than 0.9 that there is one causal SNP in the region that influences both traits (15).

*Conflict of Interest statement*. There is no conflict of interest from any of the participating co-authors.

## Supplementary Material

Supplemental_Figures_ddab197

Supplemental_Tables_final_ddab197

Supplemental_Methods_ddab197

## URLs

PhenoScanner: http://www.phenoscanner.medschl.cam.ac.uk/

GWAS Catalog: https://www.ebi.ac.uk/gwas/

Phecodes: https://phewascatalog.org/phecodes
