# Supplementary material for: Genomic and pleiotropic analyses of resting QT interval identifies novel loci and overlap with atrial electrical disorders
Source: Hum Mol Genet. 2021 Jul 19;30(24):2513–23. doi: 10.1093/hmg/ddab197 (PMC8643508; doi:10.1093/hmg/ddab197)
Supplement: Supplemental_Figures_ddab197 [file supplemental_figures_ddab197.pdf]

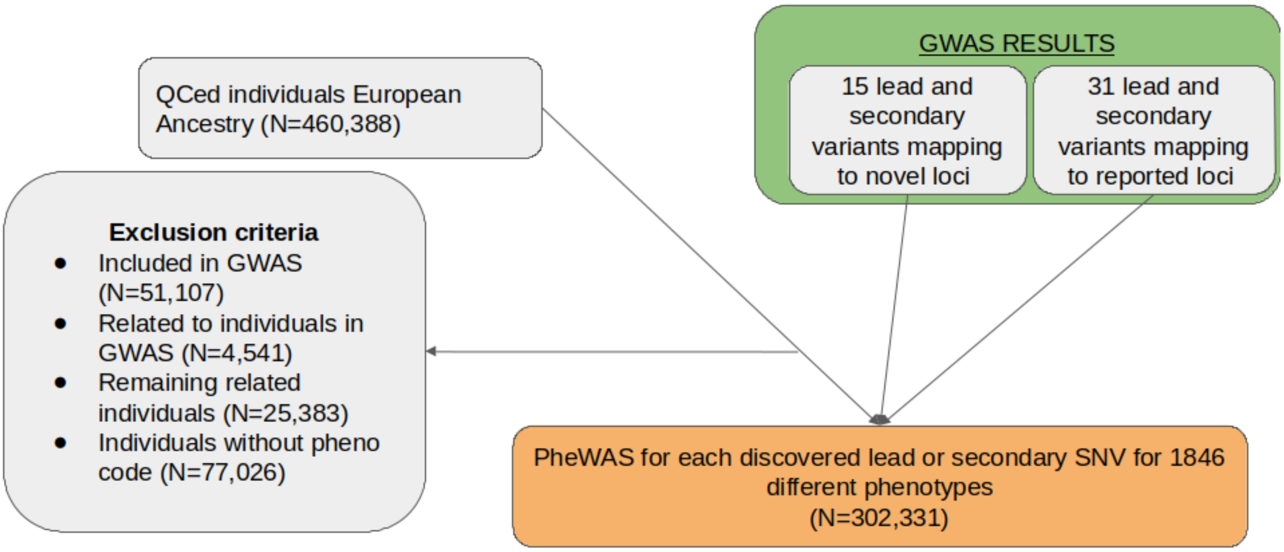


**Supplemental Fig. 1. | Design of the Phenome-wide association study (PheWAS).** We selected all European ancestry individuals from European ancestry in UK Biobank that passed genetic quality control (QC) who were not in included in or related to the GWAS cohort. All ICD9 and ICD10 diagnostic codes available from the hospital episodes statistics were converted to pheno codes. A total of 302,331 individuals with ≥1 phenocode available were included in the PheWAS including all primary and secondary variants (novel and previously reported) discovered in the full dataset GWAS.

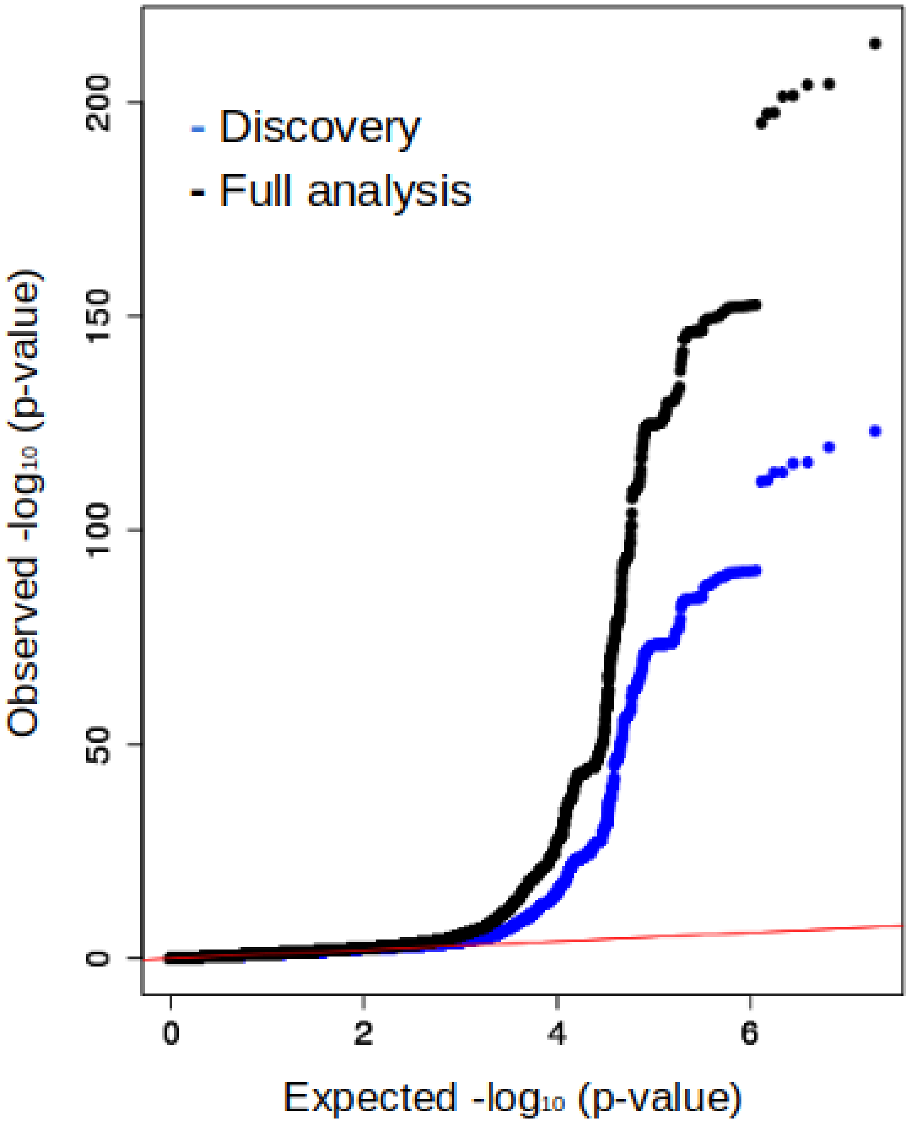


**Supplemental Fig. 2 | QQ plots**

Discovery (blue) and full cohort analysis (black). Corresponding λ values are 1.05 and 1.099, respectively.

(A)

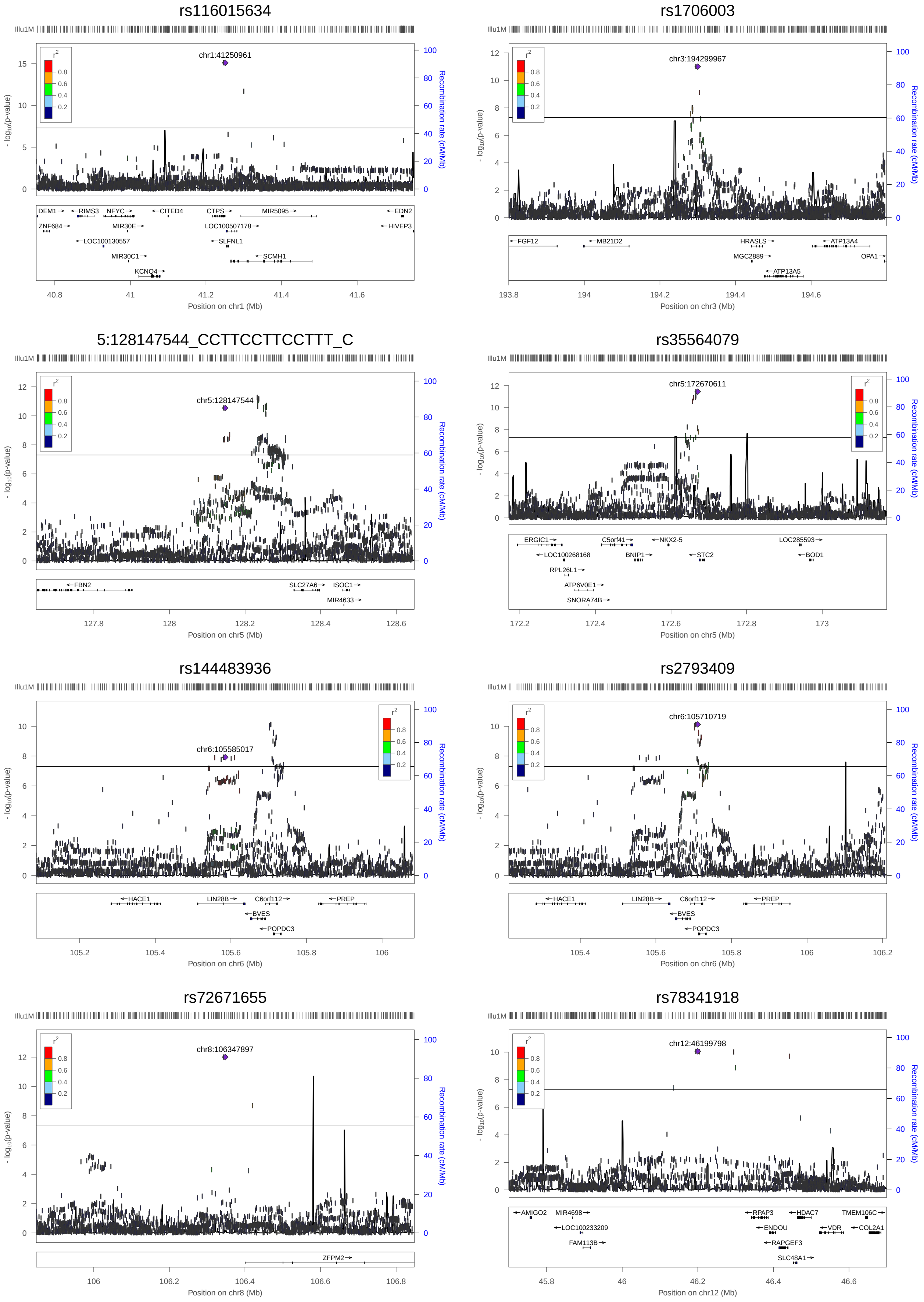
rs116015634
chr1:41250961
Position on chr1 (Mb)
DEM1
RIMS3
NFYC
CITED4
CTPS
MIR5095
EDN2
ZNF684
MIR30E
LOC100507178
HIVEP3
LOC100130557
SLFNL1
MIR30C1
SCMH1
KCNQ4
rs1706003
chr3:194299967
Position on chr3 (Mb)
FGF12
MB21D2
HRASLS
ATP13A4
MGC2889
OPA1
ATP13A5
5:128147544_CCTTCCTTCCTTT_C
chr5:128147544
Position on chr5 (Mb)
FBN2
SLC27A6
ISOC1
MIR4633
rs35564079
chr5:172670611
Position on chr5 (Mb)
ERGIC1
C5orf41
NKX2-5
LOC285593
LOC100268168
BNIP1
STC2
BOD1
RPL26L1
ATP6V0E1
SNORA74B
rs144483936
chr6:105585017
Position on chr6 (Mb)
HACE1
LIN28B
C6orf112
PREP
BVES
POPDC3
rs2793409
chr6:105710719
Position on chr6 (Mb)
rs72671655
chr8:106347897
Position on chr8 (Mb)
ZFPM2
rs78341918
chr12:46199798
Position on chr12 (Mb)
AMIGO2
MIR4698
RPAP3
HDAC7
TMEM106C
LOC100233209
ENDOU
VDR
COL2A1
FAM113B
RAPGEF3
SLC48A1
- log10(p-value)
Recombination rate (cM/Mb)
Illu1M
r2

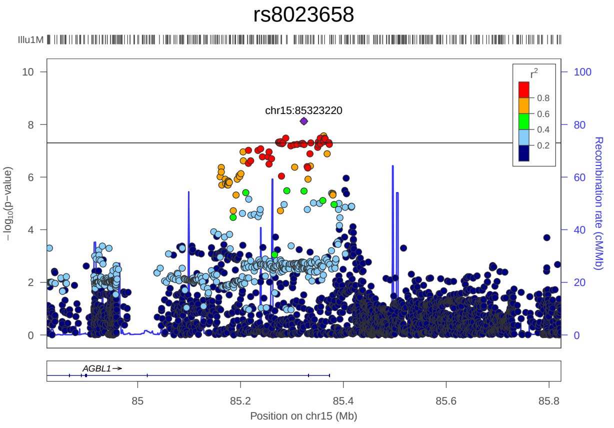
rs8023658
Illu1M
chr15:85323220
r2
0.8
0.6
0.4
0.2
−log10(p−value)
Recombination rate (cM/Mb)
AGBL1→
85
85.2
85.4
85.6
85.8
Position on chr15 (Mb)

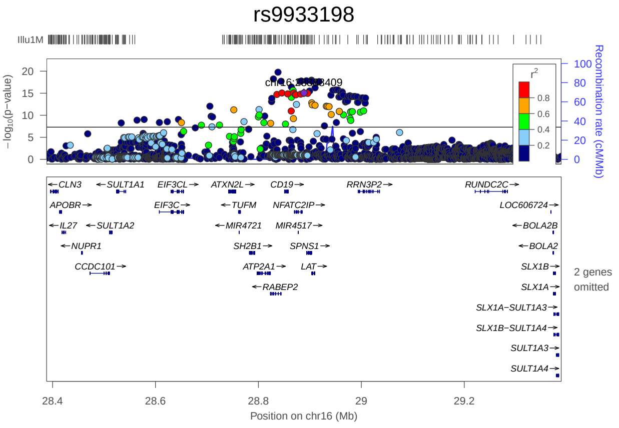
rs9933198
Illu1M
r2
0.8
0.6
0.4
0.2
−log10(p−value)
Recombination rate (cM/Mb)
←CLN3
←SULT1A1
EIF3CL→
ATXN2L→
CD19→
RRN3P2→
RUNDC2C→
APOBR→
EIF3C→
←TUFM
NFATC2IP→
LOC606724→
←IL27
←SULT1A2
←MIR4721
MIR4517→
←BOLA2B
←NUPR1
SH2B1→
SPNS1→
←BOLA2
CCDC101→
ATP2A1→
LAT→
SLX1B→
←RABEP2
SLX1A→
SLX1A−SULT1A3→
SLX1B−SULT1A4→
SULT1A3→
SULT1A4→
2 genes omitted
28.4
28.6
28.8
29
29.2
Position on chr16 (Mb)

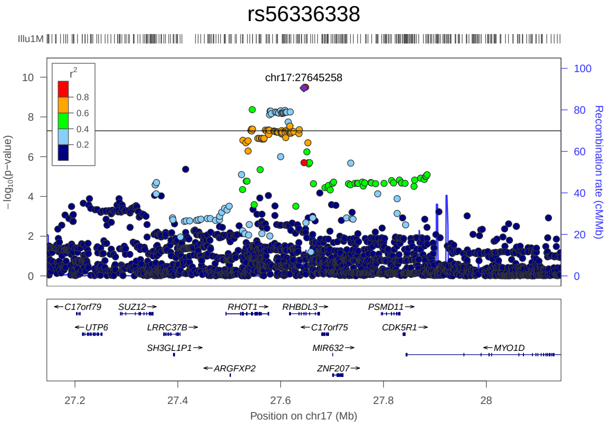
rs56336338
Illu1M
chr17:27645258
r2
0.8
0.6
0.4
0.2
−log10(p−value)
Recombination rate (cM/Mb)
←C17orf79
SUZ12→
RHOT1→
RHBDL3→
PSMD11→
←UTP6
LRRC37B→
←C17orf75
CDK5R1→
SH3GL1P1→
MIR632→
←MYO1D
←ARGFXP2
ZNF207→
27.2
27.4
27.6
27.8
28
Position on chr17 (Mb)

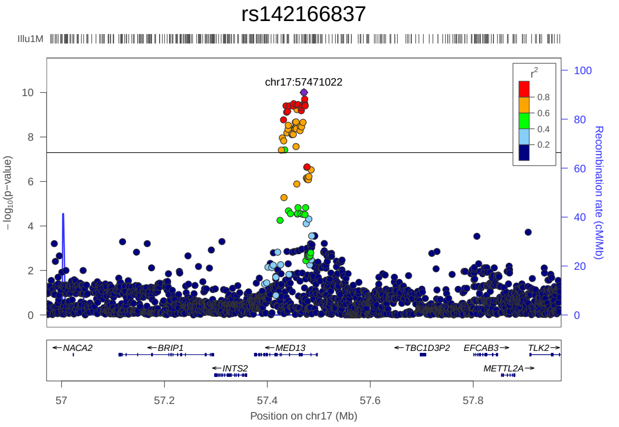
rs142166837
Illu1M
chr17:57471022
r2
0.8
0.6
0.4
0.2
−log10(p−value)
Recombination rate (cM/Mb)
←NACA2
←BRIP1
←MED13
←TBC1D3P2
EFCAB3→
TLK2→
←INTS2
METTL2A→
57
57.2
57.4
57.6
57.8
Position on chr17 (Mb)

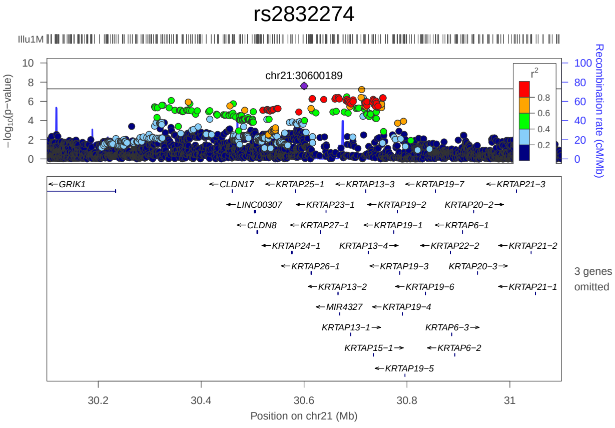
rs2832274
Illu1M
chr21:30600189
r2
0.8
0.6
0.4
0.2
−log10(p−value)
Recombination rate (cM/Mb)
←GRIK1
←CLDN17
←KRTAP25−1
←KRTAP13−3
←KRTAP19−7
←KRTAP21−3
←LINC00307
←KRTAP23−1
←KRTAP19−2
KRTAP20−2→
←CLDN8
←KRTAP27−1
←KRTAP19−1
←KRTAP6−1
←KRTAP24−1
KRTAP13−4→
←KRTAP22−2
←KRTAP21−2
←KRTAP26−1
KRTAP19−3
KRTAP20−3→
←KRTAP13−2
←KRTAP19−6
←KRTAP21−1
←MIR4327
←KRTAP19−4
KRTAP13−1→
KRTAP6−3→
KRTAP15−1→
←KRTAP6−2
←KRTAP19−5
3 genes omitted
30.2
30.4
30.6
30.8
31
Position on chr21 (Mb)

(B)

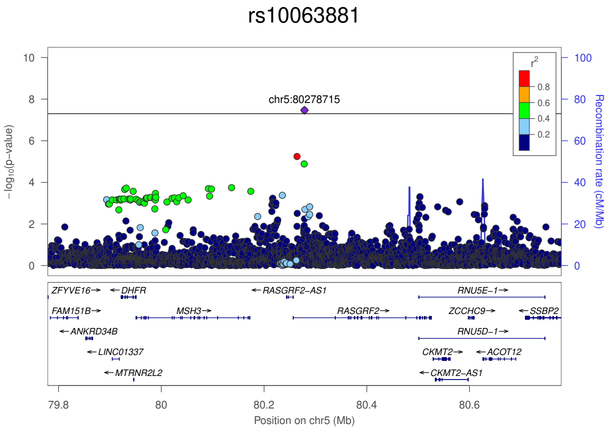


(C)

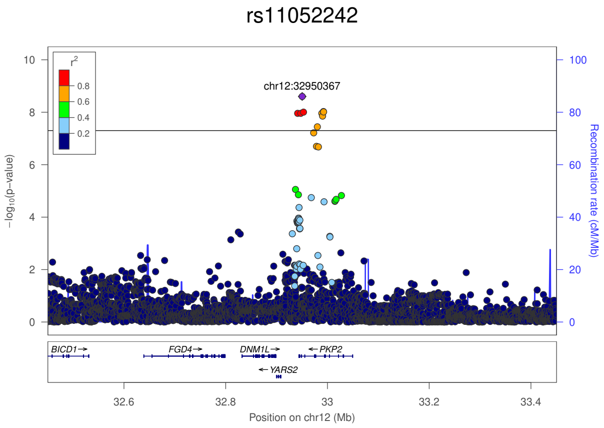


**Supplemental Fig. 3. | Regional visualisation of genome-wide association scan results for novel loci discovered in the combined-sex (A) and sex-stratified analyses (B: Men, C: Women).**

**(A)**

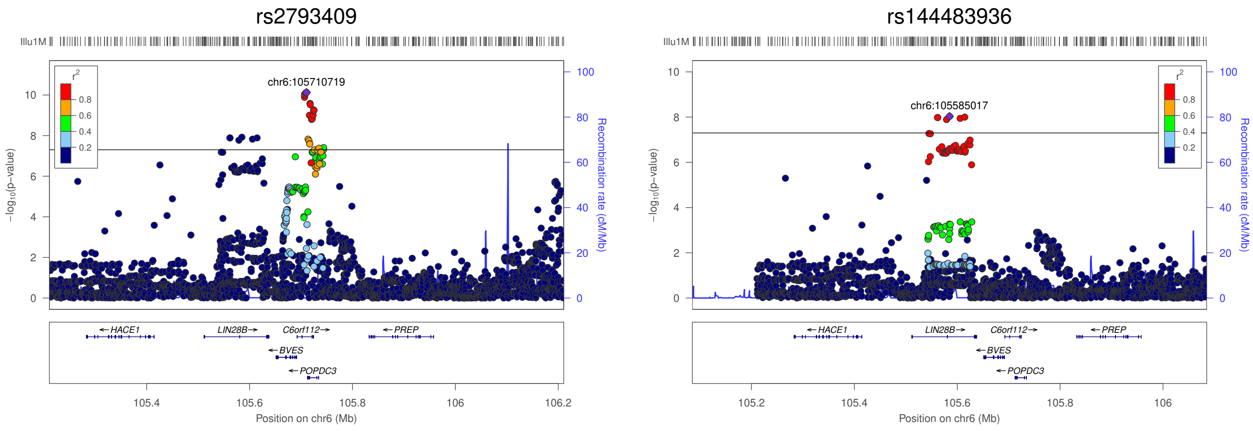

rs2793409
chr6:105710719
HACE1
LIN28B
C6orf112
PREP
BVES
POPDC3
Position on chr6 (Mb)
−log10(p−value)
Recombination rate (cM/Mb)
r2
rs144483936
chr6:105585017


**(B)**

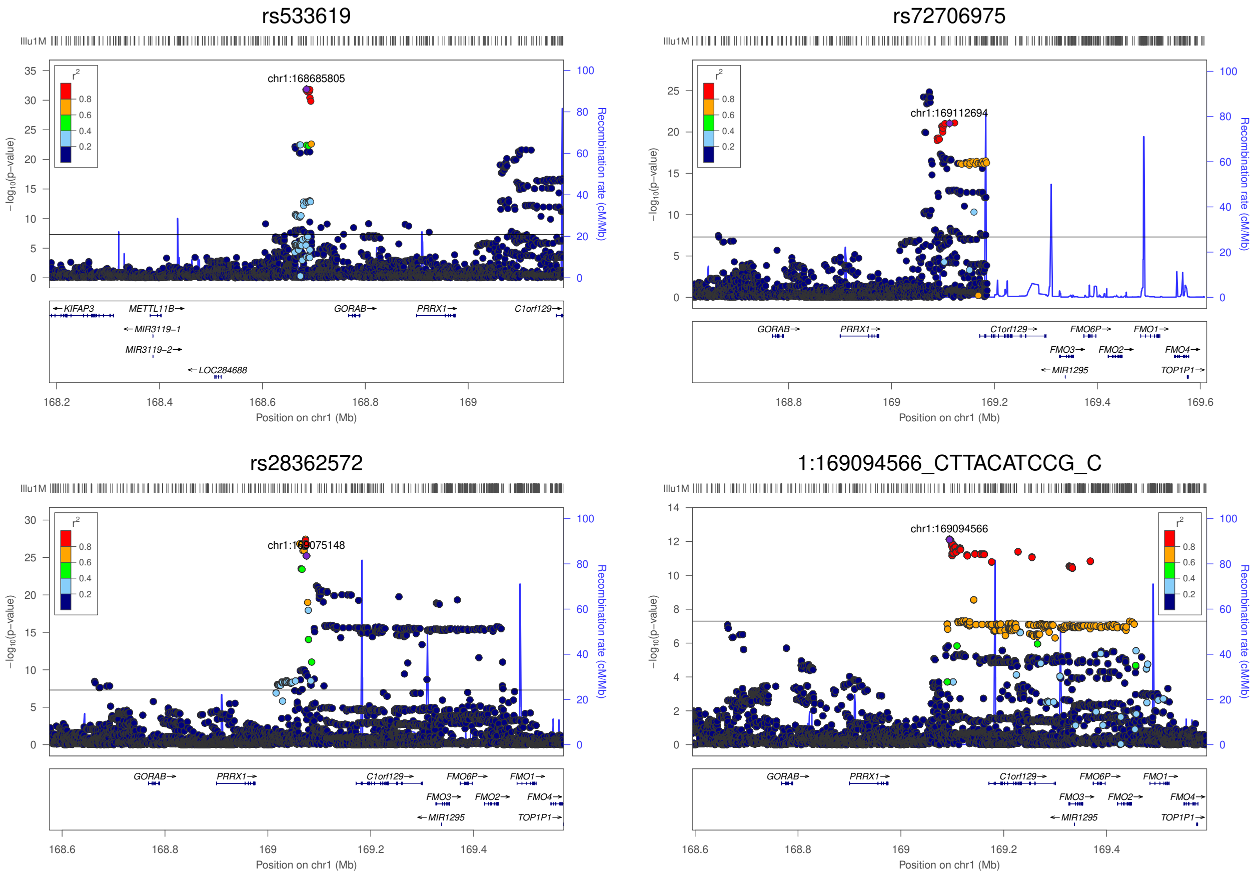

rs533619
chr1:168685805
KIFAP3
METTL11B
MIR3119−1
MIR3119−2
LOC284688
GORAB
PRRX1
C1orf129
rs72706975
chr1:169112694
FMO6P
FMO1
FMO3
FMO2
FMO4
MIR1295
TOP1P1
rs28362572
chr1:169075148
1:169094566_CTTACATCCG_C
chr1:169094566
Position on chr1 (Mb)
−log10(p−value)
Recombination rate (cM/Mb)
r2

**(C)**

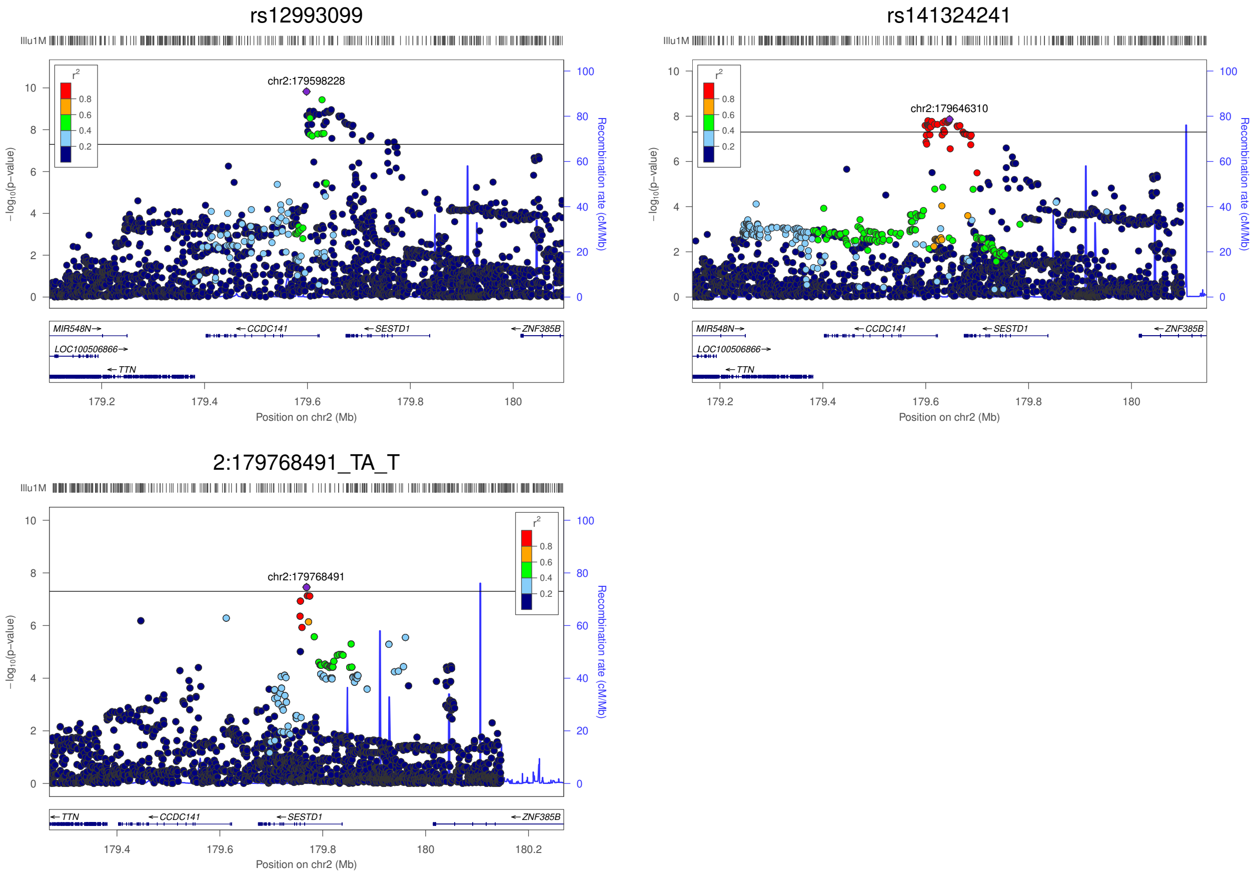


**Supplemental Fig. 4. | Regional visualisation of secondary signals before & after conditional analysis for novel *PREP* locus (A), and previously reported DPT (B) and *TTN* (C) loci**

**Supplemental Fig. 5 | Functional element Overlap analysis of the Results of Genome Wide Association Study using Functional element Overlap analysis of the Results of GWAS Experiments (FORGE).** Results show enrichment within DNase I–hypersensitive sites that are transcriptionally active in fetal heart samples.

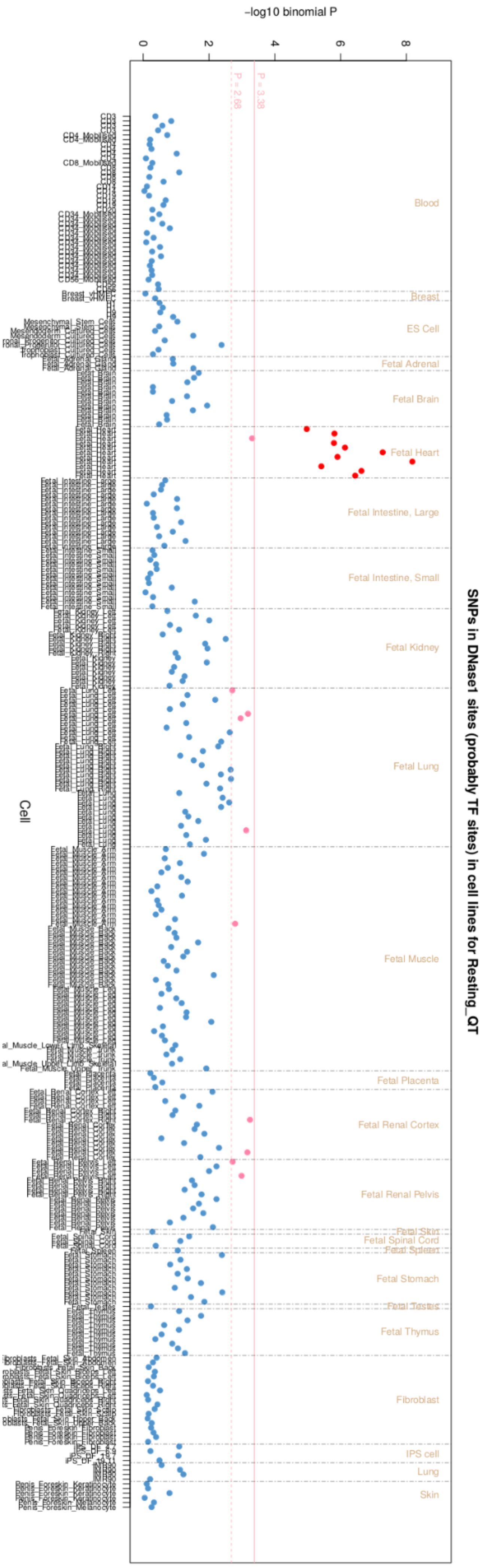

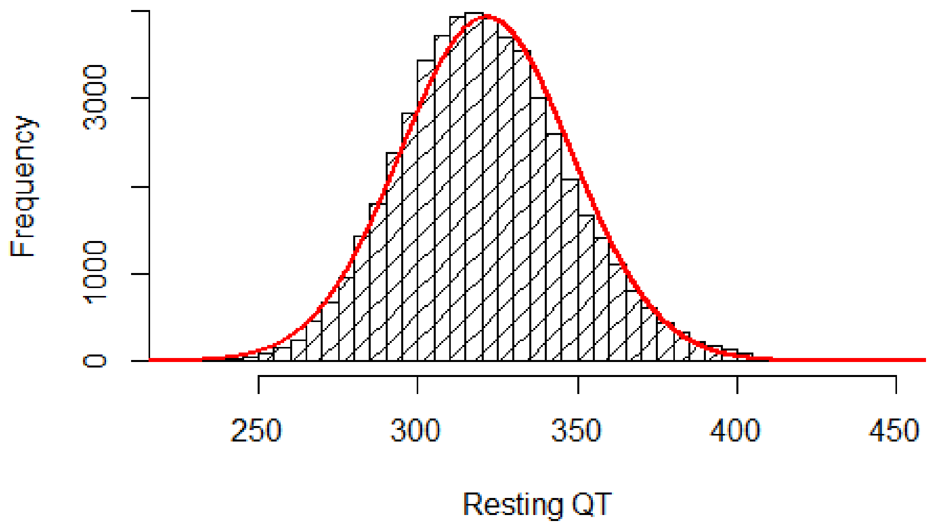


**Supplemental Fig. 6. | Distribution of the resting QT interval (ms) in 52,107 individuals from UK Biobank.**
