# Supplementary material for: Genomic and pleiotropic analyses of resting QT interval identifies novel loci and overlap with atrial electrical disorders
Source: Hum Mol Genet. 2021 Jul 19;30(24):2513–23. doi: 10.1093/hmg/ddab197 (PMC8643508; doi:10.1093/hmg/ddab197)
Supplement: Supplemental_Methods_ddab197 [file supplemental_methods_ddab197.pdf]

Supplemental Material

Supplemental Methods

Genetic quality control

Genotyping was performed by UKB using the Applied Biosystems UK BiLEVE Axiom Array or the UKB AxiomTM Array(1). SNVs were imputed centrally by UKB using the Haplotype Reference Consortium (HRC) and UK10K/1000Genomes haplotype resource panels(1). We applied genetic quality control (QC) to exclude individuals with bad genotype quality, provided by UKB, i.e. high missingness or heterozygosity and discordance between the self-reported sex, and the sex inferred from the genotypes were excluded (N = 2,655) (1). We then restricted our dataset to individuals of European ancestry only (N = 71,713). This was achieved using the k-means function in R as a clustering algorithm to select clusters according to information from the first two principal components (PC1 and PC2). The k-means algorithm partitions the points into k groups such that the sum of squares from points to the assigned cluster centres is minimised. Then, we applied k-means separately to cluster according to each of PC1 and PC2, and initially only with k=4, for a 4-way clustering, to correspond to the 4 main ethnic clusters within UKB: White, African, Asian and Chinese. We then created an overall clustering, according to the intersections of the PC1-4means-clustering and the PC2-4means-clustering, so that participants were only categorised as "White" overall, if they were contained in the "White" cluster for both PC1 and PC2. Next, we created an overall "Mixed / Other" cluster, for any participants, whose clustering differed between PC1 and PC2. Finally, we combined the PCA ancestry clusters with the self-reported ethnicity. Individuals were only included if the results PCA-clustering results matched the self-reported ancestry.

Genome wide association analyses

Model SNVs were selected from the genotyped SNVs, required for the subsequent GWASs using PLINK 1.9(2). This selection was based on the following criteria: a minor allele frequency (MAF) > 1%, a Hardy-Weinberg equilibrium (HWE) with a threshold of *P*-value = $1x10^{-6}$, and missingness < 0.0015. Using the model SNVs and ~ 9 million imputed variants with MAF ≥ 1%, and imputation quality (INFO) > 0.3. GWASs were performed to discover SNVs associated with QT dynamics during exercise and recovery using a linear mixed model method (BOLT-LMM)(3) under the additive genetic model including ~ 9 million imputed SNVs with MAF ≥ 1% and INFO > 0.3. We included sex, diabetes, age, body mass index (BMI), genotyping array (binary indicator: UKB vs. UK BiLEVE) and resting RR interval..

Since we did not have access to an independent study that could serve as a replication study, we randomly divided our dataset into discovery (N ~ 30,000) and replication (N ~ 22,000) datasets and removed individuals with kinship coefficient > 0.088. We compiled all SNVs with $P < 1 \times 10^{-6}$ from the discovery analysis and mapped them to individual loci based on genomic distance of > 500 Kb to each side of the lead SNV. If multiple SNVs fit the selection criteria for a single region, only the SNV with the smallest P value was considered for follow up. As a QC step, for each trait we reviewed the selected SNVs to check for unrealistically high effect sizes, large standard errors, and none were observed. Regional association plots were produced for all selected SNVs and these were carefully reviewed. For each trait, replication was confirmed if the P-value in the replication cohort was lower than the Bonferroni threshold and the effect size was in the same direction observed in discovery analyses in the replication cohort.

In addition to the replication analyses, we also performed a full dataset GWAS for the three traits. Additional loci, considering one lead SNV per 1 Mb region, for each trait reaching a genome-wide significance threshold ($P \leq 5 \times 10^{-8}$) from the full dataset GWAS

were identified. In addition to the replication study, we also performed a GWAS in the full datasets and sex-stratified GWASs to identify additional loci.

Sex-stratified analyses

We performed sex-stratified analyses in the full cohort (27,612 women and 24,495 man), with the same covariates in the regression model as the full dataset analyses but excluding sex. We then looked for genetic variants that were significantly associated with any of the traits in one of the sex-specific cohorts, but not in the primary analysis. For each variant we also calculated the significance of the difference between the men-specific and women-specific effect sizes (β men, β women) with corresponding standard errors SE*men* and SE*women* using the t statistic(4):

$$t = \frac{b_{men} - b_{women}}{\sqrt{SE_{men}{}^2 + SE_{women}{}^2 - 2r \cdot SE_{men} \cdot SE_{women}}}$$

References

1. Bycroft, C., Freeman, C., Petkova, D., et al. (2018) The UK Biobank resource with deep phenotyping and genomic data. *Nature*, **562**, 203–209.
2. Purcell, S., Neale, B., Todd-Brown, K., et al. (2007) PLINK: A tool set for whole-genome association and population-based linkage analyses. *Am. J. Hum. Genet.*
3. Loh, P. R., Bhatia, G., Gusev, A., et al. (2015) Contrasting genetic architectures of schizophrenia and other complex diseases using fast variance-components analysis. *Nat. Genet.*, **47**, 1385–1392.
4. Randall, J. C., Winkler, T. W., Kutalik, Z., et al. (2013) Sex-stratified Genome-wide Association Studies Including 270,000 Individuals Show Sexual Dimorphism in Genetic Loci for Anthropometric Traits. *PLoS Genet.*, **9**.
